# An Analysis of Cardiac Disorders Associated With Chimeric Antigen Receptor T Cell Therapy in 126 Patients: A Single-Centre Retrospective Study

**DOI:** 10.3389/fonc.2021.691064

**Published:** 2021-06-14

**Authors:** Kunming Qi, Zhiling Yan, Hai Cheng, Wei Chen, Ying Wang, Xue Wang, Jiang Cao, Huanxin Zhang, Wei Sang, Feng Zhu, Haiying Sun, Depeng Li, Qingyun Wu, Jianlin Qiao, Chunling Fu, Lingyu Zeng, Zhenyu Li, Junnian Zheng, Kailin Xu

**Affiliations:** ^1^ Blood Diseases Institute, Affiliated Hospital of Xuzhou Medical University, Xuzhou Medical University, Xuzhou, China; ^2^ Department of Hematology, Affiliated Hospital of Xuzhou Medical University, Xuzhou, China; ^3^ Key Laboratory of Bone Marrow Stem Cell, Jiangsu Province, Xuzhou, China; ^4^ Cancer Institute, Xuzhou Medical University, Xuzhou, China

**Keywords:** CAR-T cell therapy, cardiac disorders, CRS, corticosteroids, tocilizumab

## Abstract

**Introduction:**

Chimeric antigen receptor T (CAR-T) cells are effective in treating hematological malignancies. However, in patients receiving CAR-T therapy, data characterizing cardiac disorders are limited.

**Methods:**

126 patients with hematologic malignancies receiving CAR-T cell therapy were analyzed to determine the impact of CAR-T therapy on occurrence of cardiac disorders, including heart failure, arrhythmias, myocardial infarction, which were defined by the Common Terminology Criteria for Adverse Events (CTCAE). Parameters related to cardiac disorders were detected including myocardial enzyme, NT-proBNP and ejection fraction (EF). Cardiovascular (CV) events included decompensated heart failure (HF), clinically significant arrhythmias and CV death.

**Results:**

The median age of patients was 56 years (6 to 72 years). 58% patients were male, 62% had multiple myeloma, 20% had lymphoma and 18% had ALL. 33 (26%) patients had cardiac disorders, most of which were grade 1-2. 13 patients (10%) were observed with cardiac disorders grade 3-5, which comprised 5(4%) patients with new-onset HF, 2 (2%) patients with new-onset arrhythmias, 4 (3%) patients with the acute coronary syndrome, 1(1%) patient with myocardial infarction and 1(1%) patient with left ventricular systolic dysfunction. There were 9 CV events (7%) including 6 decompensated heart failure, 1 clinically significant arrhythmias and 2 CV deaths. Among the 33 patients with cardiac disorders, the patients with cardiac disorders CTCAE grade 3-5 had higher grade CRS (grade ≥ 3) than those with cardiac disorders CTCAE grade ≤ 2 (*P <*0.001). More patients with cardiac disorders CTCAE grade 3-5 were observed in the cohort who did not receive corticosteroids and/or tocilizumab therapy timely comparing with those who received corticosteroids and/or tocilizumab therapy timely (*P* =0.0004).

**Conclusions:**

Cardiac disorders CAR-T cell therapy were common and associated with occurrence of CRS. However, most cases were mild. For patients with CRS grade 3-5, timely administration of corticosteroids and/or tocilizumab can effectively prevent the occurrence and progression of cardiac disorders.

## Introduction

Chimeric antigen receptor (CAR)-T cell therapy has sparked a wave of optimism in relapsed/refractory hematologic malignancies. CAR-T cells targeting new tumor antigens would emerge in the near future. Some side effects were observed after CAR-T cell therapies with cytokine releasing syndrome (CRS) as the most common one, presenting as fever, hypotension, hypoxia, and capillary leakage. Neurotoxicity and coagulation dysfunction have also been reported (1–4). These side effects were intensively studied, however, other side effects such as cardiac disorders might be underestimated. Case report has shown some cases of cardiac disorders (5). Thus, a comprehensive study is needed to evaluate occurrence of cardiac disorders after CAR-T cell therapy.

Here, we performed a comprehensive analysis of the incidence, dynamic changes and outcomes of cardiac disorders defined by CTCAE in 126 patients with relapsed/refractory (R/R) hematologic malignancies after receiving CAR-T cell therapy. The correlation between cardiac disorders and CRS was also analyzed.

## Subject and Methods

### Study Design and Patient Selection

The study cohort was derived from the patients receiving CAR-T at Affiliated Hospital of Xuzhou Medical University between January 1, 2019, and November 20, 2020; 126 patients were included. (China) (Clinical Trials: NCT02782351, NCT03207178, ChiCTR-OIC-17011272). All patients were followed-up until a fixed calendar date (i.e., January 31, 2021). Clinical events were extracted by detailed chart review. For the patients who died during the follow-up, the last follow-up date was the date of death. This was a retrospective study conducted according to the Declaration of Helsinki’s principles with approval by the Ethics Committee of the Affiliated Hospital of Xuzhou Medical University. Informed consents were obtained from all patients.

### CAR-T Cell Manufacturing and Infusion

The humanized single-chain variable fragment (scFv) sequence specific for CD19 was derived from clone FMC63 as previously described (6) and the anti-CD20, anti-CD22 and anti-BCMA scFv was derived from a murine anti-human CD20, CD22 and BCMA monoclonal antibody. The scFv sequence for CD19, CD20 and BCMA were inserted in tandem with the human CD8 transmembrane, CD8 hinge, 4-1BB costimulatory domain, CD3z intracellular regions, and T2A-EGFRt sequence. The scFv sequence for CD22 was inserted in tandem with the human CD8 transmembrane, CD8 hinge, CD28 costimulatory domain, CD3z intracellular regions, and T2A-EGFRt sequence. CARs targeting CD19, CD20, CD22 and BCMA were synthesized and subcloned into lentivirus expression vector Lenti-EF1a-puro and stably expressed in CD3-positive T cells after transfection of lentiviral vector.

Peripheral blood mononuclear cells were obtained from patients by leukapheresis as previously described (3). Most patients received lymphodepletion chemotherapy with the FC regimens included both fludarabine (750 mg/m^2^, day-5) and cyclophosphamide (30 mg/m^2^/d, days -5~2). On day 0, patients with MM received CD19 CAR-T cell and BCMA CAR-T cell infusion at the median dose 2 × 10^6^ cells/kg (1.4 - 4 × 10^6^ cells/kg), patients with B-NHL received CD19 CAR-T cell and CD22 CAR-T cell infusion at the median dose 2 × 10^6^ cells/kg (0.8-6 × 10^6^ cells/kg) and patients with ALL received a single dose of CD19 CAR-T cell infusion at the median dose 1 × 10^6^ cells/kg (0.8-2 × 10^6^ cells/kg) respectively.

### Collection of Clinical and Laboratory Data

Peripheral blood was collected before lymphodepletion, on day -3 or day -1, and at approximately 1~3, 4~6, 7~10, 11~13, 14~16, 17~20, 21-24, 25~30, 31-40, 41-50 days after CAR-T cell infusion for analysis of complete blood counts, hepatic function, renal function, hs-cTnT, NT-proBNP, cytokine including IL-6, Ferritin, CRP, IL-8, IFN-γ and IL-10. The morphological characteristics of bone marrow were evaluated on day 0, 14 and 28 respectively. Minimal residual disease was detected by flow cytometry. If the patient did not die, the CAR-T cells were followed up for at least 90 days.

### Cardiac Disorders Diagnosis and CRS Grading

Cardiac disorders was defined according to Common Terminology Criteria for Adverse Events (CTCAE; version 4.03) (7). According to the hs-cTnT assay, the lower limit of detection is 3 ng/L for the 99th percentile is 14 ng/L according to the manufacturer. The diagnosis of myocardial infarction required a rise and/or fall of hs-cTnT with at least one value above the 99th percentile upper reference limit, with the symptoms of ischaemia or development electrocardiogram (8). NT-proBNP elevation of patients ≤ 50 years old, patients > 50 years old and patients with baseline glomerular filtration rate (GFR) < 60ml/min were defined > 450 pg/ml, > 900 pg/ml and >1200 pg/ml respectively. A reduction in left ventricular ejection fraction (LVEF) was defined as a reducing at least ten percentage points, less than 50%. Cardiovascular events included decompensated HF, clinically significant arrhythmias and CV death (9). CRS was graded according to consensus criteria proposed by Lee et al. (10). All results were reviewed and confirmed by the research team without considering other variables. The onset of CRS was defined as the first appearance of a fever after CAR-T therapy, excluding other factors for fever. The time to corticosteroid or tocilizumab administration was defined as the time from the onset of CRS to the administration of corticosteroid or tocilizumab. This article reports cardiac disorders presenting within 50 days after the first CAR-T cell infusion.

### Statistical Analysis

Descriptive statistics (median/interquartile range [IQR], count, and percentage) are reported for key variables. Continuous data were compared using unpaired Student’s *t*-tests and categorical data were compared using the chi-square or the Fisher exact test. The factors associated with cardiac disorders were explored using ordinal regression with cardiac disorders CTCAE frequency as the dependent variable and baseline glomerular filtration rate, sex, age, diagnosis, underlying disease (including diabetes, hypertension, etc) and CRS as independent variables. The Wilcoxon test was adopted for continuous variables between 2 groups, and the Kruskal-Wallis test was for multiple groups. The Spearman correlation test was used for correlation analysis. Statistical significance was defined using a 2-tailed *p*-value < 0.05. Statistical analyses were performed using IBM SPSS for Windows 25.0 software (SPSS, Inc., Chicago, IL).

## Results

### Patients’ Baseline Characteristics

Baseline demographics and clinical characteristics of the 126 patients with R/R hematologic malignancies receiving lymphodepletion chemotherapy and CAR-T cell therapy, are shown in **Table 1**. In the entire cohort, the median age was 56 years (range, 6 to 72 years), including 73(58%) males and 53(42%) females. The most of patients were multiple myeloma (MM) (*n* = 78, 62%), followed by non-Hodgkin’s lymphoma (NHL) (*n* = 25, 20%) and acute lymphoblastic leukemia (ALL) (*n*=23, 18%). In the MM cohort, 13 patients (86%) had low baseline glomerular filtration rate (< 60ml/min) and 44(57%) patients bone marrow plasma cells were ≥10%. In the NHL cohort, 21 patients (84%) were Ann Arbor stage III~IV. The percentage of bone marrow blasts was ≥20% in 13 (57%) patients with ALL. Before the CAR-T cell therapy, 120 (95%) patients received lymphodepletion chemotherapy of FC regimen, and 6 (5%) received non-FC regimen, including 2 with fludarabine alone, 1 with cyclophosphamide alone and 3 without regimen (**Table 1**).

**Table 1:** Baseline characteristics and factors associated with cardiac disorders.

Cardiac disorders CTCAE grade		Grade 0^a^	Grade 1-2^a^	Grade 3-5^a^	Total	Univariate^b^	Multivariable^c^
Overall, n (%)		93 (74)	20 (16)	13 (10)	126(100)		
Age, n (%)	<40 years	19 (83)	1 (4)	3 (13)	23	0.413	
	40–60 years	48 (75)	10 (16)	6 (9)	64		
	>60 years	26 (67)	9 (23)	4 (10)	39		
Sex, n (%)	Male	55 (75)	15 (21)	3 (4)	73	0.012	0.116
	Female	38 (72)	5 (9)	10 (19)	53		
Diagnosis	MM^d^	53 (68)	16 (20)	9 (12)	78	0.274	
n (%)	NHL^e^	22 (88)	2 (8)	1 (4)	25		
	ALL^f^	18 (78)	2 (9)	3 (13)	23		
ECOG	0	62 (77)	11(14)	7 (9)	80	0.461	
	1-2	31(67)	9 (20)	6 (13)	46		
Primary refractory^g^		6 (60)	2 (20)	2 (20)	10	0.805	
Prior lines of therapy	2-4	38 (73)	9 (17)	5 (10)	52		
	>4	49 (77)	9 (14)	6 (9)	64		
Baseline	≤14ng/L	93 (75)	19 (15)	12 (10)	124	–	
hs-cTnT	>14ng/L	0 (0)	1 (50)	1 (50)	2		
Baseline	Normal	92 (78)	17 (14)	9 (8)	118	–	
NT-proBNP	Increased^h^	1 (12)	3 (38)	4 (50)	8		
Baseline	<60ml/min	4 (29)	4 (29)	6 (42)	14	<0.0001	0.031
Glomerular	≥60ml/min	89 (80)	16 (14)	7 (6)	112		
Filtration rate							
BM plasma	<10%	25 (74)	7 (20)	2 (6)	34	0.378	
cells of MM	≥10%	28 (64)	9 (20)	7 (16)	44		
Ann Arbor	I-II	4 (100)	0 (0)	0 (0)	4	–	
stage of NHL	III-IV	18 (86)	2 (9)	1 (5)	21		
BM Blast	<20%	10 (100)	0 (0)	0 (0)	10	–	
of ALL	≥20%	8 (62)	2 (15)	3 (23)	13		
Lymphodepletion	FC	88(73)	20 (17)	12 (10)	120	–	
Regimen^i^, n(%)	Non-FC	5 (83)	0 (0)	1(17)	6		
CAR-T	0.8-2	66 (72)	15 (16)	11 (12)	92	0.57	
Cell Dose	3-6	27 (79)	5 (15)	2 (6)	34		
Charlson	≦2	30 (77)	4 (10)	5 (13)	39	0.386	
Comorbidity	2-4	49 (75)	12 (19)	4 (6)	65		
Index^j^	>4	14 (64)	4 (18)	4 (18)	22		
Diabetes	Yes	3 (33)	4 (45)	2 (22)	9	–	
	No	90 (77)	16 (14)	11 (9)	17		
Hypertension	Yes	15 (65)	6 (26)	2 (9)	23	0.333	
	No	78 (76)	14 (14)	11 (10)	3		
Coronary artery	Yes	1 (100)	0 (0)	0 (0)	1	–	
Disease	No	92 (74)	20 (16)	13 (10)	125		
Chronic Heart	Yes	0 (0)	0 (0)	2 (100)	2	–	
Failure	No	93 (75)	20 (16)	11 (9)	124		
Atrial fibrillation	Yes	0 (0)	0 (0)	1 (100)	1	–	
	No	93 (74)	20 (16)	12 (10)	125		
CRS^k^, n (%)	Grade 0	23 (100)	0 (0)	0 (0)	23	<0.0001	<0.0001
	Grade 1-2	69(85)	12 (15)	0 (0)	81		
	Grade 3-5	1 (5)	8 (36)	13 (59)	22		

a Percentages are shown in parentheses.

b Two-sided P values calculated based on the Mann-Whitney test for continuous variables and based on chi-square or the Fisher exact test for categorical variables.

c Ordinal regression models were performed to assess the impact of baseline factors on the occurrence of cardiac disorders.

d multiple myeloma.

e non-Hodgkin’s lymphoma.

f acute lymphoblastic leukemia.

g Refractory was defined as disease progression on or within 60 days after the last dose of the most recent drug given in each drug class.

h NT-proBNP elevation of patients ≤ 50 years old, patients > 50 years old and patients with baseline glomerular filtration rate < 60ml/min were defined > 450 pg/ml, > 900 pg/ml

and >1200 pg/ml respectively.

i FC regimens included both fludarabine (750 mg/m^2^, day-5) and cyclophosphamide (30 mg/m^2^/d, days -5～-2).

j Charlson Comorbidity Index was scored according to consensus criteria proposed by Charlson M et al. (11) (**Supplementary Table 1**).

k cytokine release syndrome.

### Factors Associated With Subsequent Cardiac Disorders

Within 50 days of CAR-T cell infusion, cardiac disorders of any grade were more frequent in female patients (*P* = 0.012), the patients with low baseline glomerular filtration rate (< 60ml/min) (*P* < 0.0001) and the patients with CRS (*P* < 0.0001) by univariate analyses (**Table 1**). The patient’s age, diagnosis, ECOG, the number of plasma cells in bone marrow of MM patients, Charlson Comorbidity Index and hypertension were not associated with cardiac disorders by univariate analyses. The baseline of hs-cTnT, NT-proBNP, Ann Arbor stage of NHL, ALL patients with a high tumor burden (blasts ≥20% in bone marrow), lymphodepletion regimens and underlying diseases (including diabetes, coronary artery disease, chronic heart failure and atrial fibrillation) were no statistical significance. Multivariable analysis showed that after CAR-T cell therapy, the patients with low baseline glomerular filtration rate (< 60ml/min) and CRS were associated with an increased risk of cardiac disorders (*P* = 0.031, *P* < 0.0001 perspective) (**Table 1**).

### Cardiac Disorders and CRS After CAR-T Cell Immunotherapy

**Figure 1:**
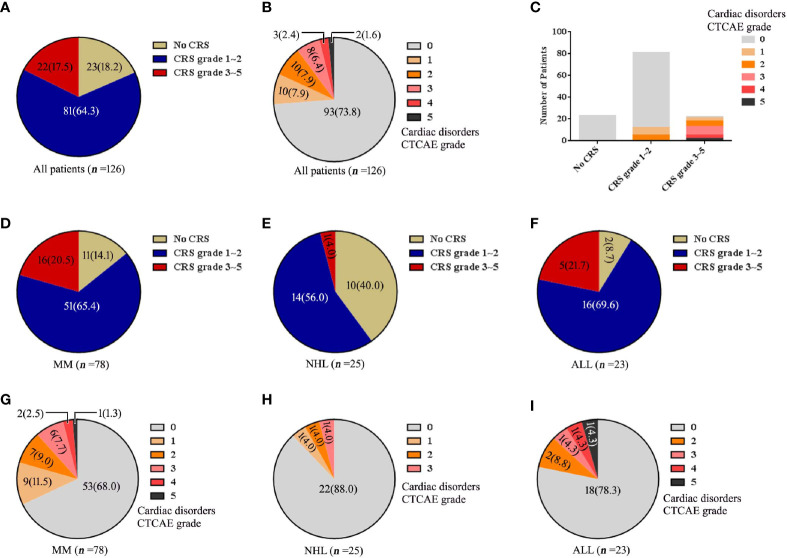
The changes of CRS, cardiac disorders CTCAE before and after CAR-T cell infusion. **(A)** Numbers of patients with each CRS. **(B)** Numbers of patients with each cardiac disorders grade. **(C)** Numbers of patients with each grade of cardiac disorders and CRS. The numbers of patients with each CRS grade are shown for each disease of **(D)** MM, **(E)** NHL and **(F)** ALL. Cardiac disorders CTCAE for each disease of **(G)** MM, **(H)** NHL and **(I)** ALL. Percentage is shown in parentheses.

Of 126 patients treated with lymphodepletion chemotherapy and CAR-T cell infusion, 33(26%) patients (25 MM patients, 3 NHL patients, 5 ALL patients) had cardiac disorders as defined by the Common Terminology Criteria for Adverse Events (CTCAE). There were 10 patients (8%) with CTCAE grade 1, 10 patients (8%) with CTCAE grade 2, 8 patients (6%) with CTCAE grade 3, 2 patients (2%) with CTCAE grade 4 and 3 patients (2%) with CTCAE grade 5. In MM cohort, cardiac disorders CTCAE 1, CTCAE 2, CTCAE 3, CTCAE 4 and CTCAE 5 were 9, 7, 6, 2 and 1 patients. In NHL cohort, cardiac disorders CTCAE 1, CTCAE 2, CTCAE 3, CTCAE 4 and CTCAE 5 were 1, 1, 1, 0 and 0 patients respectively. In ALL cohort, cardiac disorders CTCAE 1, CTCAE 2, CTCAE 3, CTCAE 4 and CTCAE 5 were 0, 2, 1, 1, 1 patients respectively (**Figure 1**).

CRS was found in all 33 patients (26%) with cardiac disorders. Among 20 (16%) patients with cardiac disorders CTCAE grade 1-2, there were 12 patients with CRS grade 1-2 and 8 patients with CRS grade 3-5. Among 13 (10%) patients with cardiac disorders CTCAE grade 3-5, all patients had severe CRS (grade 3-5) (**Figure 1**).

Of 13 patients (10%) with cardiac disorders grade 3-5, 5(4%) patients had new-onset HF, 2 (2%) patients had new-onset arrhythmias, 4 (3%) patients had the acute coronary syndrome, 1(1%) patient had myocardial infarction and 1(1%) patient had left ventricular systolic dysfunction. There were 9 CV events (7%) with a median onset time of 5 days (IQR: 3 to 9 days) among all cardiac disorder patients. The CV events included 6 decompensated heart failures, 1 clinically significant arrhythmias and 2 CV deaths (**Table 2**).

**Table 2:** 33 (26%) patients in the entire cohort of 126 had cardiac disorders.

Cardiac disorders CTCAE	Grade 1^a^	Grade 2^a^	Grade 3^a^	Grade 4^a^	Grade 5^a^	Total^a^
Heart failure^b^	7 (6)	3 (2)	3 (2)	2 (2)	0 (0)	15 (12)
New Arrhythmia^c^	3 (2)	2 (2)	1 (1)	0 (0)	1( 1)	7 (6)
Acute coronary syndrome	–	5 (4)	4 (3)	0 (0)	0 (0)	9 (7)
Myocardial disease^d^	0 (0)	0 (0)	0 (0)	0 (0)	1 (1)	1 (1)
Left ventricular systolic dysfunction	–	–	0 (0)	1 (1)	0 (0)	1 (1)
New Valve disease^e^	0 (0)	0 (0)	0 (0)	0 (0)	0 (0)	0 (0)
Pericardial disease^f^	0 (0)	0 (0)	0 (0)	0 (0)	0 (0)	0 (0)
Cardiac arrest	0 (0)	0 (0)	0 (0)	0 (0)	0 (0)	0 (0)

a Percentage is shown in parentheses.

b New onset of heart failure, including left ventricular failure and right ventricular dysfunction.

c Asystole, Atrial fibrillation, Atrial flutter, Atrioventricular block complete, Atrioventricular block first degree, Mobitz (type) II atrioventricular block, Mobitz type I, Paroxysmal atrial tachycardia, sick sinus syndrome, Sinus bradycardia, Sinus tachycardia, Supraventricular tachycardia, Ventricular arrhythmia, Ventricular fibrillation and Ventricular tachycardia.

d Myocardial infarction, Myocarditis and Restrictive cardiomyopathy.

e Tricuspid valve disease, Pulmonary valve disease, Mitral valve disease and Aortic valve disease.

f Constrictive pericarditis, Pericardial effusion, Pericardial tamponade and Pericarditis.

### Severe Cardiac disorders Are More Frequent in Patients With Severe CRS

**Figure 2:**
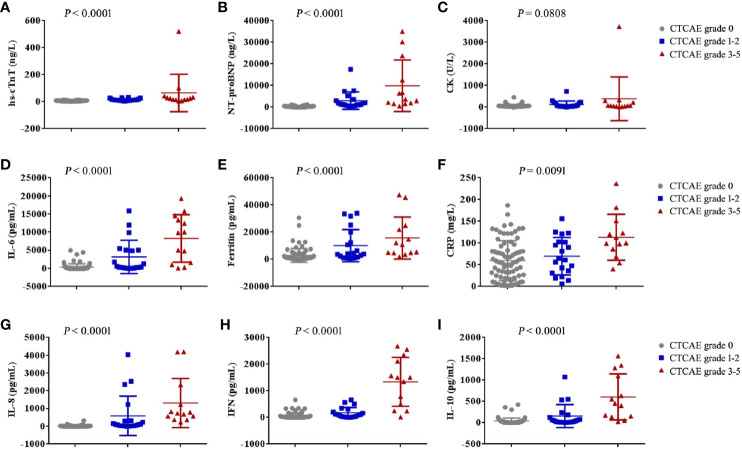
The maximum values of myocardial enzyme parameters, NT-proBNP and cytokines in patients with cardiac disorders CTCAE grade 0, 1-2 and 3-5 after CAR-T cell infusion. The maximum hs-cTnT **(A)**, maximum NT-proBNP **(B)**, maximum CK **(C)**, maximum IL-6 **(D)**, maximum Ferritin **(E)**, maximum CRP **(F)**, maximum IL-8 **(G)**, maximum IFN-γ **(H)** and maximum IL-10 **(I)** in the first 30 days after CAR-T cell infusion are shown in patients who had cardiac disorders CTCAE grade 0 (n = 80), cardiac disorders CTCAE grade 1-2 (n = 20) and cardiac disorders CTCAE grade 3-5 (n =13). Each point represents data from a single patient. The median and IQR are shown. Two-sided P values were determined using the Kruskal-Wallis test.

The patients who developed CTCAE grade ≥3 cardiac disorders had more severe CRS (p < 0.001, **Tables  1**, **3**). The occurrence time of CRS grade 3-5 was earlier than that of cardiac disorders CTCAE grade 1-2 and 3-5. The median onset time of CRS grade 3-5 and cardiac disorders CTCAE grade 3-5 was 3 days (IQR: 1 to 7 days) and 8 days (IQR: 4 to 9 days) (*P* = 0.0054) (**Table 4**). Earlier onset of CRS after CAR-T therapy was associated with a higher risk of subsequent developing severe cardiac disorders. The severity of cardiac disorders was related to the higher peak concentrations of hs-cTnT, NT-proBNP, ferritin, C-reactive protein (CRP) and multiple cytokines, including IL-6, IL-8, IFN-γ and IL-10 (**Figure 2**).

**Table 3:** Cardiac disorders CTCAE grade and CRS grade.

Cardiac disorders CTCAE	CRS grade^a^ 1-2	CRS grade^a^ 3-5	Total^a^	*P*
Heart failure^b^				0.044
Grade 1-2	6 (5)	4 (3)	10( 8)	
Grade 3-5	0 (0)	5 (4)	5 (4)	
New Arrhythmias^c^				0.047
Grade 1-2	5 (4)	0 (0)	5 (4)	
Grade 3-5	0 (0)	2 (2)	2 (2)	
Acute coronary syndrome				1
Grade 1-2	1 (1)	4 (3)	5 (4)	
Grade 3-5	0 (0)	4 (3)	4 (3)	
Myocardial disease^d^				1
Grade 1-2	0 (0)	0 (0)	0 (0)	
Grade 3-5	0 (0)	1 (1)	1 (1)	
Left ventricular systolic dysfunction				–
Grade 1-2	–	–	–	
Grade 3-5	0 (0)	1 (1)	1 (1)	
New Valve diseases^e^				–
Grade 1-2	0 (0)	0 (0)	0(0)	
Grade 3-5	0 (0)	0 (0)	0 (0)	
Pericardial diseases^f^				–
Grade 1-2	0 (0)	0 (0)	0 (0)	
Grade 3-5	0 0)	0 (0)	0 (0)	
Cardiac arrest				–
Grade 1-2	–	–	–	
Grade 3-5	0 (0)	0 (0)	0(0)	
Total				0.0005
Grade 1-2	12 (10)	8 (6)	20 (16)	
Grade 3-5	0 (0)	13 (10)	13 (10)	

^a^Percentage is shown in parentheses. P-value was tested by the Chi-Square test.

^b,c,d,e^ and ^f^ are the same as in **Table 2**.

**Table 4:** The temporal relation between cardiac disorders CTCAE and severe CRS.

	Cardiac disorders CTCAE		CRS
	Grade 1-2	Grade 3-5	Grade 3-5
Onset time (day)			
Median	9	8	3*
IQR	4-11	4-9	1-7
Range	3-25	2-23	1-10
Maximum time (day)^a^			
Median	11	14	12
IQR	7-15.75	8.5-17	8-15.25
Range	4-30	6-31	5-30
Duration time (day)^b^			
Median	6	12*	13***
IQR	3-9	8-21.5	9-24
Range	2-20	2-25	5-34
Recovery time (day)^c^			
Median	13	20*	19*
IQR	11-19	17-29	16.25-27.75
Range	6-45	8-31	7-37

a The time required from the onset time to the peak (day).

b The time from the onset to recovery (day).

c The time from the beginning of CAR-T cell infusion to return to normal (day).

*P < 0.05, *** P < 0.001.

**Figure 3:**
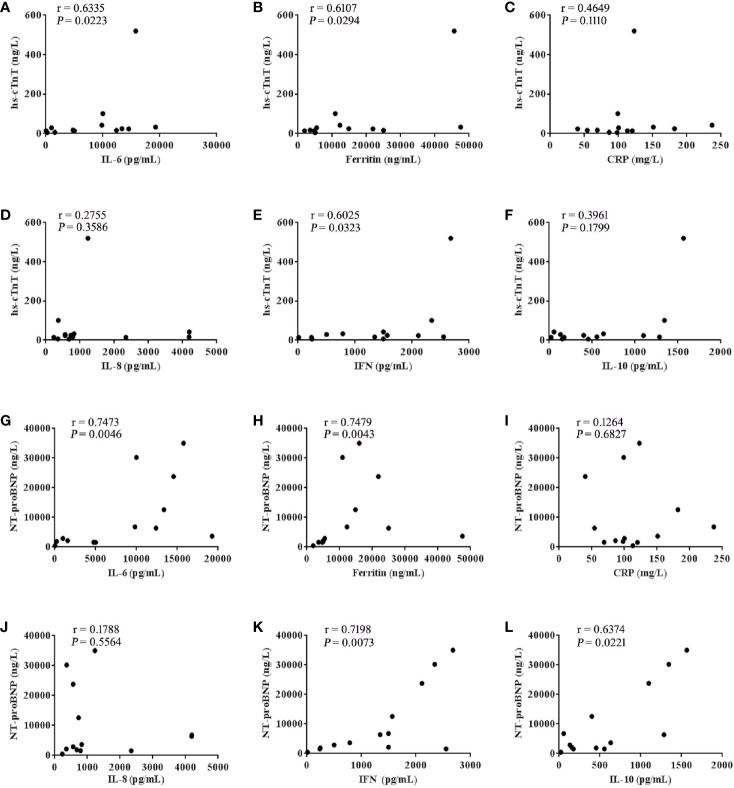
hs-cTnT and NT-proBNP were closely correlated to cytokine levels in patients with CTCAE grade 3-5 (n = 13). The correlation of hs-cTnT concentration with serum **(A)** IL-6, **(B)** Ferritin, **(C)** CRP, **(D)** IL-8, **(E)** IFN-γ and **(F)** IL-10. The correlation of NT-proBNP concentration with serum **(G)** IL-6, **(H)** Ferritin, **(I)** CRP, **(J)** IL-8, **(K)** IFN-γ and **(L)** IL-10. Each point represents data from a single patient; r values were determined using the spearman correlation test.

After further analysis of the correlation between the levels of hs-cTnT, NT-proBNP and cytokines in patients with the grade 3-5 cardiac disorders CTCAE (*n* =13), we found that the values of hs-cTnT were positively correlated with the levels of serum IL-6, Ferritin, IFN-γ (**Figures 3A, B, E**). The values of NT-proBNP were positively correlated with the levels of serum IL-6, Ferritin, IFN-γ and IL-10 (**Figures 3G, H, K, L**).

### Treatment of Cardiac Disorders and CRS

In 10 patients with cardiac disorders CTCAE grade 1, which were asymptomatic or mild, no intervention was needed. In 10 patients with CTCAE grade 2, 3 patients with HF were given torasemide and the other patients were only given symptomatic treatment. In cardiac disorders CTCAE grade≥3 cohort, all patients required an intervention that included the administration of diuretic, amiodarone, cedilanid and/or IV metoprolol and/or vasopressors and oxygen supplementation. Some patients were typically transferred to the intensive care unit for further management. After treatment, 2 (2%) patients died, among which one patient died of malignant arrhythmia and another died of myocardial infarction. However, 31 of 33 patients (94%) recovered. The arrhythmia and HF disappeared quickly after treatment. NT proBNP and hs TnT returned to normal baseline levels within one month after treatment.

**Tables 5** and **6** shows the treatments in patients with CRS (*n* = 103) and cardiac disorders. In cohort 1, patients were given corticosteroids and/or tocilizumab therapy within 24 hours after the onset of CRS and in cohort 2 patients were not given corticosteroids and/or tocilizumab therapy within 24 hours or received corticosteroids and/or tocilizumab therapy more than 24 hours after the onset of CRS. Among the 51 patients with CRS grade 1 and 30 patients with CRS grade 2, there was no difference in the incidence of cardiac disorders between cohort 1 and cohort 2 (*P* = 0.1336, 0.3742). While among the 22 patients with grade 3-5, 2 of 8 patients (25%) in cohort 1 developed cardiac disorders, which was lower than 11 of 14 patients (79%) in cohort 2 (*P* = 0.0260). In 33 patients with cardiac disorders, compared with 11 of 15 patients (73%) developed cardiac disorders (CTCAE Grade 3-5) in cohort 2, there were fewer patients in cohort 1, with only 2 of 18 patients (11%) progressed to cardiac disorders (CTCAE grade 3-5) (*P* = 0.0004) (**Tables 5**, **6**).

**Table 5:** The relationship between CRS grade 1-2, cardiac disorders and intervention therapy.

Cardiac disorders CTCAE	Grade 0	Grade 1-2	Total	*P*
CRS grade 1	47 (92)	4 (8)	51 (100)	
Cohort 1^a^	26 (87)	4 (13)	30	0.1336
Cohort 2^b^	21 (100)	0 (0)	21	
CRS grade 2	22 (73)	8 (27)	30 (100)	
Cohort 1^a^	14 (67)	7 (33)	21	0.3742
Cohort 2^b^	8 (89)	1 (11)	9	

Data were described as n (%). P-value was tested by the Chi-Square test.

a Patients were given corticosteroids and/or tocilizumab therapy within 24 hours after the onset of CRS.

b Patients were not given corticosteroids and/or tocilizumab therapy within 24 hours or received corticosteroids and/or tocilizumab therapy more than 24 hours after the onset of CRS.

**Table 6:** The relationship between CRS grade 3-5, cardiac disorders and intervention therapy.

Cardiac disorders CTCAE	Grade 0-2	Grade 3-5	Total	*P*
CRS grade 3-5	9 (41)	13 (59)	22 (100)	
Cohort 1^a^	6 (75)	2 (25)	8	0.026
Cohort 2^b^	3 (21)	11 (79)	14	
Cardiac disorders				
CTCAE	20 (61)	13 (39)	33 (100)	
Cohort 1^a^	16 (89)	2 (11)	18	0.0004
Cohort 2^b^	4 (27)	11 (73)	15	

Data were described as n (%). P-value was tested by the Chi-Square test.

a Patients were given corticosteroids and/or tocilizumab therapy within 24 hours after the onset of CRS.

b Patients were not given corticosteroids and/or tocilizumab therapy within 24 hours or received corticosteroids and/or tocilizumab therapy more than 24 hours after the onset of CRS.

## Discussion

### Early Detection of Severe Cardiac Disorders Is Necessary

CRS is one of the most common side effects in CAR-T cell therapy (12–14). Other toxicities including neurotoxicity and coagulation dysfunction were also reported (2–4). However, there have been very few reports of cardiac disorders after CAR-T. Cardiac disorders had different manifestations, of which the most common was HF (12%), followed by acute coronary syndrome (7%) and arrhythmia (6%), and the least was myocardial infarction (1%). The diagnosis of CAR-T cell-related cardiac disorders required the appearance of new symptoms or signs of HF, arrhythmia or myocardial infarction. Although most cardiac disorders are mild and temporary, some patients developed severe cardiac disorders, which may be life-threatening in the form of malignant arrhythmias, myocardial infarction, and decompensated HF. Early diagnosis and treatment of CAR-T-related cardiac disorders is an important step to reduce the mortality after CAR-T cell therapy. Serial testing of myocardial enzymes, especially hs-cTnT and NT-proBNP, is very necessary for early detection of cardiac disorders. Alvi RM (9) reported that an elevated troponin occurred in 29 of 53 tested patients (54%) and was associated with an increased risk for a CV event. In our study, 9 and 12 of 13 patients with cardiac disorders (CTCAE grade 3-5) had elevated hs-cTnT and NT-proBNP, with the median peak value 23.60 ng/L (IQR: 14.00 to 37.52 ng/L) and 3657 pg/ml (IQR: 1724 to 18186 pg/ml) respectively, suggesting that continuous testing of hs-cTnT and NT-proBNP may be of use for identifying high-risk patients with cardiac disorders after CAR-T. Shalabi H also found out troponin and proBNP may help to earlier identify those patients at highest risk of severe cardiac systolic dysfunction (15).

### Risk Factors Associated With Cardiac Disorders CTCAE

Some study had demonstrate that ≈10% of patients develop cardiomyopathy in the context of high-grade CRS after CAR-T cell therapy (16). In our study, baseline factors, including patients with CRS and low baseline GFR levels were associated with an increased risk of subsequent cardiac disorders by multivariable analysis. According to univariate and multivariable analysis, patients with baseline GFR < 60ml/min were more likely to have cardiac disorders than that with baseline GFR ≥ 60ml/min. Lefebvre B also found that baseline creatinine and CRS grade 3 or 4 were independently associated with major adverse cardiovascular events (17). However, after CAR-T treatment, if the treatment were effective, many patients’ renal function would be improved with creatinine and glomerular filtration rate (GFR) returning to normal, which is consistent with our previous published paper (18). Alvi RM showed that troponin elevation was associated with CRS, but not with CAR-T type, cancer type, therapies before CAR-T, race or age (9). The Baseline of NT-proBNP was higher in patients with renal dysfunction, which was prone to heart failure (19). In our study, the baseline of hs-cTnT and NT-proBNP were no statistical significance.

### CRS Was Closely Related to Cardiac Disorders, or Cardiac Disorders Was a Part of CRS

There was a graded relationship between CRS and cardiac disorders. After CAR-T treatment, fever is the most common sign of CRS (2). In our study, 100% of patients with CAR-T-related cardiac disorders had CRS. Patients who developed cardiac disorders CTCAE grade ≥3 were more frequently observed in the severe CRS (grade ≥3) cohort (**Tables 1**, **3**) and no severe CRS (grade ≥3) was found in patients without cardiac disorders, which indicated that there was a close and graded relationship between the development of CRS and cardiac disorders. We found the occurrence time of CRS grade 3-5 was earlier than that of cardiac disorders grade 3-5, and the median onset time of CRS grade 3-5 and cardiac disorders CTCAE grade 3-5 was 3 days (IQR: 1 to 7 days) and 8 days (IQR: 4 to 9 days) (**Table 4**), suggesting that CRS may cause cardiac disorders. Some studies have found that CRS after CAR-T therapy was related to hepatic dysfunction, coagulation abnormality and neurotoxicity (20–22). Our study indicated that CRS was closely related to cardiac disorders, or cardiac disorders was a part of CRS.

### The Pathogenesis of Cardiac Disorders May Be Related to the Injury of Endothelial by a Large Number of Cytokines

Endothelial dysfunction and hypercoagulability are considered to be the main factors of CAR-T toxicity. Indeed, the biomarkers of endothelial cell activation, such as angiopoietin 2, the angiopoietin-2 to angiopetin-1 ratio and von Willebrand Factor (VWF) were higher in patients with neurotoxicity (grade ≥4) (2). Similarly, it should be noted that endothelial dysfunction is considered an early event in the pathophysiology of cardiovascular disease (23). In our study, after CAR-T therapy, cytokines such as IL-6, IFN-γ which can cause endothelial injury, were significantly increased in patients with severe cardiac disorders. We also observed that patients with earlier peak concentrations of IL-6, IFN-γ had a higher grade of cardiac disorders, suggesting that the rising rate of serum cytokine concentration as well as the peak concentration, may be determinants of the severity of cardiac disorders. IL-6, Ferritin and CRP are the most frequently detected cytokines in CRS (24, 25). After further analysis of the correlation between the levels of myocardial enzyme parameters and cytokines in patients with cardiac disorders (grade ≥3), we found the values of both hs-cTnT and NT-proBNP were positively correlated with the levels of serum IL-6, Ferritin and IFN-γ (**Figure 3**). Like IL-6, IFN-γ is a proinflammatory cytokine involved in the biological process of inducing macrophages to secrete tumor necrosis factor (TNF-α) and stimulating macrophages to release reactive oxygen species (26, 27). Thus, we speculate that besides targeting IL-6, targeting IFN-γ may be a novel strategy for managing CRS or cardiac disorders that are caused by CAR-T, although this theory requires further investigation.

### The Onset Time of CRS and Administration Time of Corticosteroids/Tocilizumab Were Associated With Severity of CRS and Cardiac Disorders

Among the 103 patients with CRS administered different treatments, we found that there was no difference in the incidence of cardiac disorders of patients with CRS grade 1-2 between cohort 1 and cohort 2. However, patients with CRS grade 3-5 in cohort 2 were more likely to develop cardiac disorders than those in cohort 1. In other words, when severe CRS (grade 3-5) occurs, prompt treatment (given corticosteroids and/or tocilizumab therapy within 24 hours) could reduce the incidence of cardiac disorders (**Tables 5**, **6**).

Tocilizumab, an antagonistic IL-6R mAb, effectively ameliorates fever and hypotension in most patients developed severe CRS after CAR-T cell (28). Our study found that in addition to IL-6, other cytokines such as IFN-γ, CRP, ferritin, IL-8 and IL-10 were significantly increased in the occurrence of cardiac disorders. Corticosteroids may be a double-edged sword in the management of immunomodulation and inflammatory response. Its pharmacological mechanisms involve reducing inflammatory cytokines production, leukocyte infiltration and phagocytosis at the onset of inflammation (29, 30). Besides, the corticosteroid can interfere with the interaction between proinflammatory factors (31, 32). Although corticosteroids may cause injury to the infused CAR-T cell and reduce the curative effect, previous studies and our results show that corticosteroids are still very important for the treatment of cytokine storm-related diseases as hemophagocytic syndrome and CRS (33–35). Our study suggested that timely administration of corticosteroids and/or tocilizumab at the early stage of CRS grade 3-5 may relieve syndromes and delay cardiac disorders progression.

### Study Limitations

These data are obtained retrospectively. As with all retrospective studies, there may be missing data at some points after CAR-T infusion. For example, some patients with CRS grade 1-2 had mild symptoms, and sometimes may miss cytokine assay. Meanwhile, because the hs-cTnT and NT-proBNP assay in our study was often based on clinical suspicion of cardiac injury (not a protocol driven study as expected), with no symptoms of heart failure, acute coronary syndrome, some patients were not given hs-cTnT or NT-proBNP assay and color doppler echocardiography examination. Therefore, the value of hs-cTnT or NT-proBNP at some time points were missed. In addition, some patients undergo cardiac MRI or ECT examinations at designated locations after developing severe cardiac disorders. This part of the data was also unavailable and without a cardiac biopsy, the exact mechanism of cardiac disorders cannot be determined.

## Conclusion

In summary, cardiac disorders were common events after CAR-T cell therapy closelyassociated with CRS. However, the most cardiac disorders were mild. Patients who developed cardiac disorders CTCAE grade ≥3 had more severe CRS (≥3 grade). The values of hs-cTnT were positively correlated with the levels of serum IL-6, Ferritin, IFN-γ. The values of NT-proBNP were positively correlated with the levels of serum IL-6, Ferritin, IFN-γ and IL-10. The onset time of CRS and administration time of corticosteroids/tocilizumab were associated with severity of CRS and cardiac disorders.

## Data Availability Statement

The raw data supporting the conclusions of this article will be made available by the authors, without undue reservation.

## Ethics Statement

The studies involving human participants were reviewed and approved by The Ethics Committee of the Xuzhou Medical University. Written informed consent to participate in this study was provided by the participants’ legal guardian/next of kin.

## Author Contributions

KQ, ZY, and HC designed the research, analyzed the data and drafted the paper. WC, YW, XW, JC, HZ, WS, FZ, HS, and DL were mainly responsible for data collection and analysis. QW, JQ, CF, and LZ were primarily responsible for statistical analysis. KX, ZL, and JZ contributed to study design and revised the manuscript. All authors contributed to the article and approved the submitted version.

## Funding

This work was supported by the National Natural Science Foundation of China (81871263, 81930005, 82070127), Natural Science Foundation of Jiangsu Province (BK2020022348), and the Postgraduate Research & Practice Innovation Program of Jiangsu (KYCX19-2229).

## Conflict of Interest

The authors declare that the research was conducted in the absence of any commercial or financial relationships that could be construed as a potential conflict of interest.
