# Supplementary material for: An Analysis of Cardiac Disorders Associated With Chimeric Antigen Receptor T Cell Therapy in 126 Patients: A Single-Centre Retrospective Study
Source: Front Oncol. 2021 Jun 14;11:691064. doi: 10.3389/fonc.2021.691064 (PMC8237759; doi:10.3389/fonc.2021.691064)
Supplement: Supplementary file 1 [file DataSheet_1.docx]

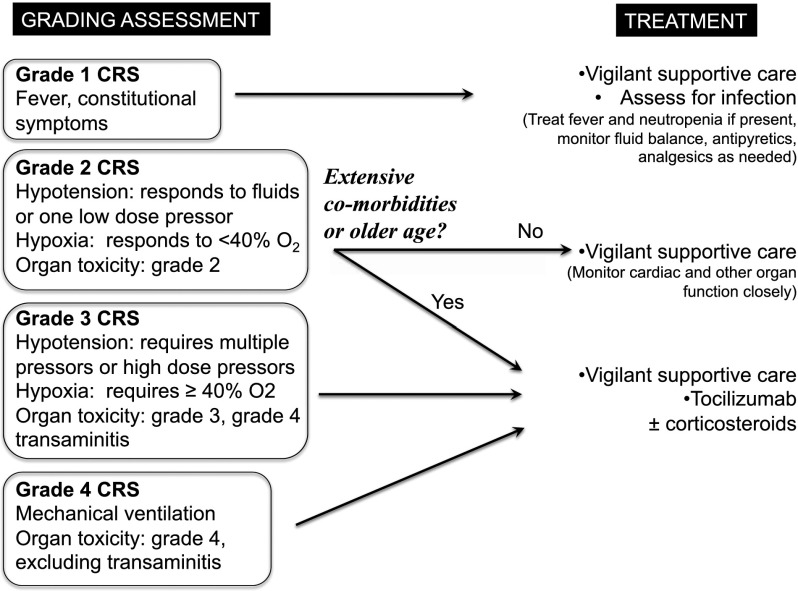


**Figure S1** Management of CRS and organ toxicity following CAR T-cell infusion.

The management of CRS is in accordance with the procedures in LEE et al. (Lee DW, Gardner R, Porter DL, et al. Blood. 2014;124:188-195).
